# Tethered heme domains in a triheme cytochrome allow for increased electron transport distances

**DOI:** 10.1002/pro.5200

**Published:** 2024-10-29

**Authors:** Benjamin W. Nash, Tomás M. Fernandes, Joshua A. J. Burton, Leonor Morgado, Jessica H. van Wonderen, Dimitri A. Svistunenko, Marcus J. Edwards, Carlos A. Salgueiro, Julea N. Butt, Thomas A. Clarke

**Affiliations:** ^1^ Centre for Molecular and Structural Biochemistry School of Biological Sciences and School of Chemistry, University of East Anglia Norwich UK; ^2^ Associate Laboratory i4HB – Institute for Health and Bioeconomy NOVA School of Science and Technology, Universidade NOVA de Lisboa Caparica Portugal; ^3^ UCIBIO – Applied Molecular Biosciences Unit, Chemistry Department NOVA School of Science and Technology, Universidade NOVA de Lisboa Caparica Portugal; ^4^ School of Life Sciences University of Essex Colchester UK

**Keywords:** cytochrome, electron transfer, flexibility, heme, microbe–mineral interface

## Abstract

Decades of research describe myriad redox enzymes that contain cofactors arranged in tightly packed chains facilitating rapid and controlled intra‐protein electron transfer. Many such enzymes participate in extracellular electron transfer (EET), a process which allows microorganisms to conserve energy in anoxic environments by exploiting mineral oxides and other extracellular substrates as terminal electron acceptors. In this work, we describe the properties of the triheme cytochrome PgcA from *Geobacter sulfurreducens*. PgcA has been shown to play an important role in EET but is unusual in containing three CXXCH heme binding motifs that are separated by repeated (PT)_x_ motifs, suggested to enhance binding to mineral surfaces. Using a combination of structural, electrochemical, and biophysical techniques, we experimentally demonstrate that PgcA adopts numerous conformations stretching as far as 180 Å between the ends of domains I and III, without a tightly packed cofactor chain. Furthermore, we demonstrate a distinct role for its domain III as a mineral reductase that is recharged by domains I and II. These findings show PgcA to be the first of a new class of electron transfer proteins, with redox centers separated by some nanometers but tethered together by flexible linkers, facilitating electron transfer through a tethered diffusion mechanism rather than a fixed, closely packed electron transfer chain.

## 1 INTRODUCTION

Many microorganisms can survive in the absence of soluble electron acceptors, such as oxygen or nitrate, by coupling the oxidation of organic molecules to the reduction of insoluble terminal acceptors in the environment (Shi et al., 2016). These insoluble acceptors include a diverse range of substrates, such as iron(III) and manganese(IV) oxides, humic acids, or carbon electrodes poised at positive potentials (Richter et al., 2012). For Gram‐negative bacteria, the reduction of insoluble substrates must occur on the extracellular face of the outer membrane. Consequently, electrons derived from the quinol pool must pass across the periplasm, through the outer membrane, and be delivered to cell surface proteins that reduce terminal electron acceptors (Gralnick & Bond, 2023; Shi et al., 2016; White et al., 2016).

These extracellular respiratory processes typically involve electron transfer through cytochromes containing multiple hemes that are assembled in the periplasm. These hemes are typically bis‐histidine coordinated and tightly packed within the protein, such that the porphyrin rings are usually ≤5 Å apart, enabling rapid electron exchange across the cytochrome (Clarke, 2022; Edwards et al., 2020; Wang et al., 2019). These cytochromes include inner membrane quinol dehydrogenases, periplasmic electron shuttles, and outer membrane porin–cytochrome complexes (White et al., 2016). *Geobacter sulfurreducens* PCA also expresses extracellular polymeric cytochrome wires that are composed of multiheme modules of 4–8 hemes (Clarke, 2022). In all these cytochromes the hemes are predicted, or experimentally shown, to be packed in tight chains.

In addition to this broad range of multiheme cytochromes required for effective respiration, *G. sulfurreducens* PCA also produces a secreted triheme cytochrome known as PgcA (Smith et al., 2014). This protein can have a significant role in the reduction of metal oxides (Tremblay et al., 2011), since a mutant *G. sulfurreducens* Δ*pgcA* strain was capable of reducing soluble iron(III) citrate but not insoluble iron(III) and manganese(IV) oxides (Zacharoff et al., 2017). Proteomic studies supported these observations by showing that PgcA was more abundant when *G. sulfurreducens* was grown in the presence of insoluble iron(III) oxides, while genomic studies showed an increase of *pgcA* expression in the presence of manganese(IV) oxides (Aklujkar et al., 2013; Ding et al., 2008).

**FIGURE 1:**
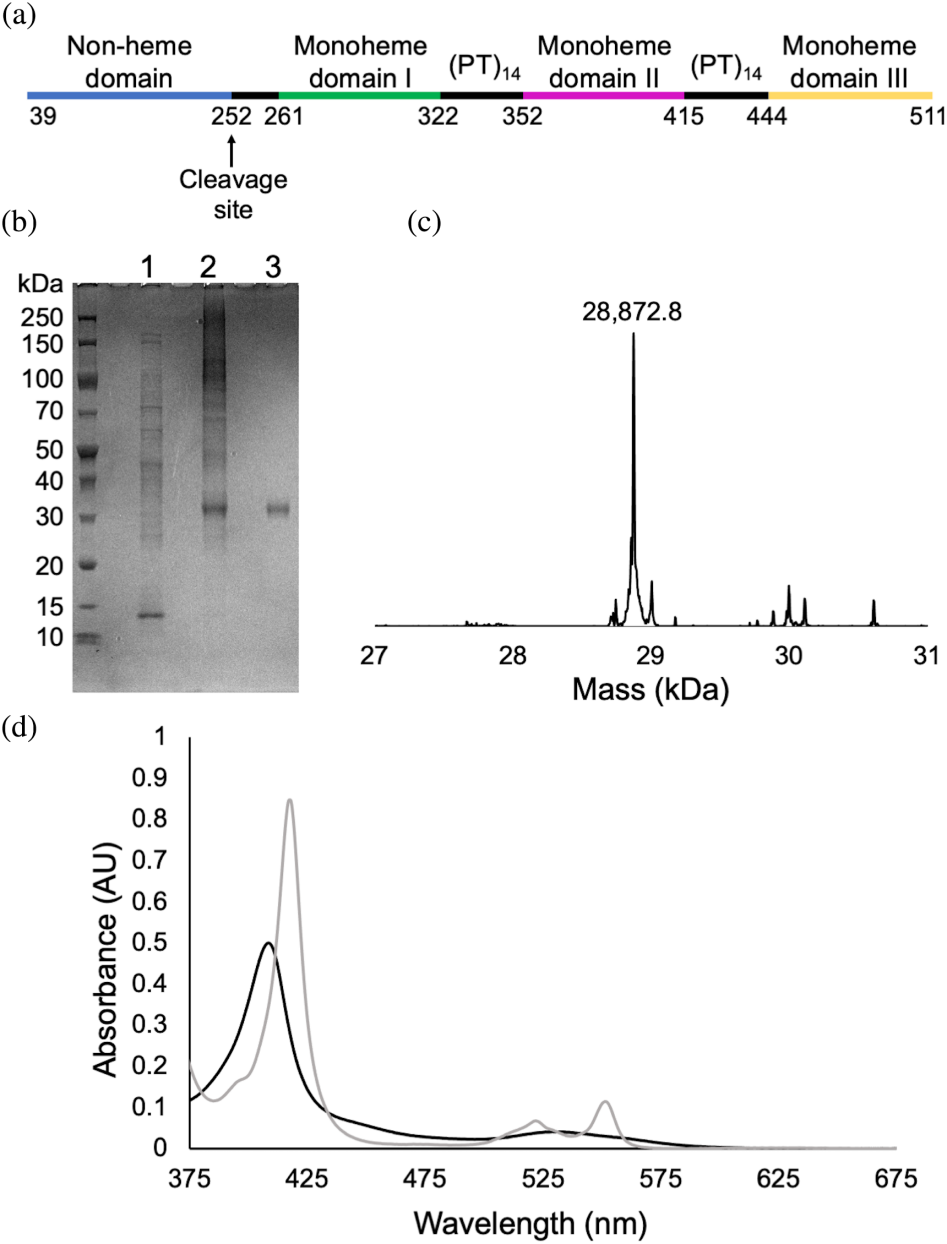
Recombinant PgcA is a triheme cytochrome containing 3 monoheme domains. (a) Domain architecture of PgcA. Cleavage at Met252 results in a triheme PgcA containing three monoheme cytochrome domains interspersed by (PT)_x_ motifs. (b) Coomassie stained gradient SDS‐PAGE gel of triheme PgcA and purification intermediates. Lane 1: clarified cell lysate; Lane 2: Strep‐Tactin affinity chromatography eluent; Lane 3: size exclusion chromatography eluent. (c) Deconvoluted mass spectra for triheme PgcA, residue 253‐C terminus fragment MW: 28,872.4 Da. (d) UV–visible spectrum of as‐purified triheme PgcA (black) in 100 mM Tris–HCl, 150 mM NaCl pH 8.0, and after reduction with excess sodium dithionite (gray).

The amino acid sequence of PgcA contains (Figure 1a) a cleavable Sec signal peptide followed by an N‐terminal lipid anchor and four domains interspersed by repeated (PT)_x_ motifs. The first domain is not predicted to contain any cofactors, while the other three domains are monoheme domains (Fernandes et al., 2023). After synthesis, PgcA is transported through the outer membrane and can be secreted into the extracellular matrix in a soluble and truncated form (Smith et al., 2014). Recombinant expression of a plasmid‐based copy of *pgcA* in *Shewanella oneidensis* resulted in the synthesis of a similarly truncated PgcA, not recognized by its type II secretion system, and trapped in the periplasm (Zacharoff et al., 2017). When a *G. sulfurreducens* strain was recently constructed with deletions of its periplasmic shuttle cytochromes PpcA‐E, it quickly adapted to compensate for their absence by developing a mutation in the PgcA N‐terminal lipid attachment motif that also trapped it in the periplasm (Choi et al., 2022). The individual monoheme domains of PgcA have recently been identified and characterized spectroscopically (Fernandes et al., 2023). The midpoint reduction potential (*E*_m_) values of each domain are similar, and NMR spectroscopy suggested that interdomain interactions were transient in nature, indicating that the protein is unlikely to transfer electrons via a rigid heme chain, as observed in other multiheme cytochromes (Fernandes et al., 2023).

Here, we report the characterization of the triheme PgcA and its constituent monoheme domains. Our findings demonstrate PgcA to be the first of a new class of redox enzymes, with redox centers separated by nanometers, but tethered together by flexible linkers and capable of electron exchange between each domain.

## 2 RESULTS

### 2.1 Heterologous expression of PgcA results in a monomeric triheme protein

Recombinant PgcA with a C‐terminal Strep‐II purification tag was isolated from *S. oneidensis* MR1 cell extracts by affinity chromatography as described in Section 4. Pooled PgcA fractions were analyzed using SDS‐PAGE and stained for heme, revealing a minor band at ~50 kDa and a major band at ~30 kDa (Figure S1a). These two heme‐containing bands both bound to the Strep‐Tactin affinity column, consistent with the isolation of two different PgcA forms, as previously observed for heterologously expressed PgcA (Zacharoff et al., 2017).

Size exclusion chromatography separated the two forms, resulting in the isolation of the ~30 kDa form of PgcA (Figure 1b). LC–MS analysis revealed a mass of 28,873 Da (Figure 1c), within 1 Da of the predicted mass for a recombinant PgcA containing three hemes of mass 615.17 Da and having undergone proteolytic cleavage between Met252 and Pro253 at the C‐terminus of the non‐heme domain (Figure 1a). The UV–visible absorbance spectrum (Figure 1d) of this triheme PgcA was consistent with an oxidized *c*‐type cytochrome, with a Soret peak at 408 nm and a spectral feature around 525 nm. The addition of sodium dithionite caused the Soret peak intensity to increase and shift to 419 nm, while the 525 nm peak separated into α‐ and β‐ bands at 530 and 550 nm, respectively, consistent with reduced *c*‐type hemes (Figure 1d). These spectral features are consistent with the average properties observed for the individual monoheme domains previously reported (Fernandes et al., 2023).

Additional plasmids containing genes encoding the monoheme PgcA domains I, II and III were produced. These constructs, called pPGCA‐DI, pPGCA‐DII and pPGCA‐DIII also included Strep‐II purification tags at their C‐terminus and the *S. oneidensis* MR‐1 MtrB signal peptide at their N‐terminus, which was previously used to overexpress recombinant cytochromes in *S. oneidensis* (Lockwood et al., 2018). The constructs were overexpressed in *S. oneidensis* MR‐1 and proteins purified as described in Section 4. The masses of these proteins were determined by LC–MS (Figure S2).

### 2.2 X‐ray crystallographic analysis of individual PgcA heme domains I, II, and III

**FIGURE 2:**
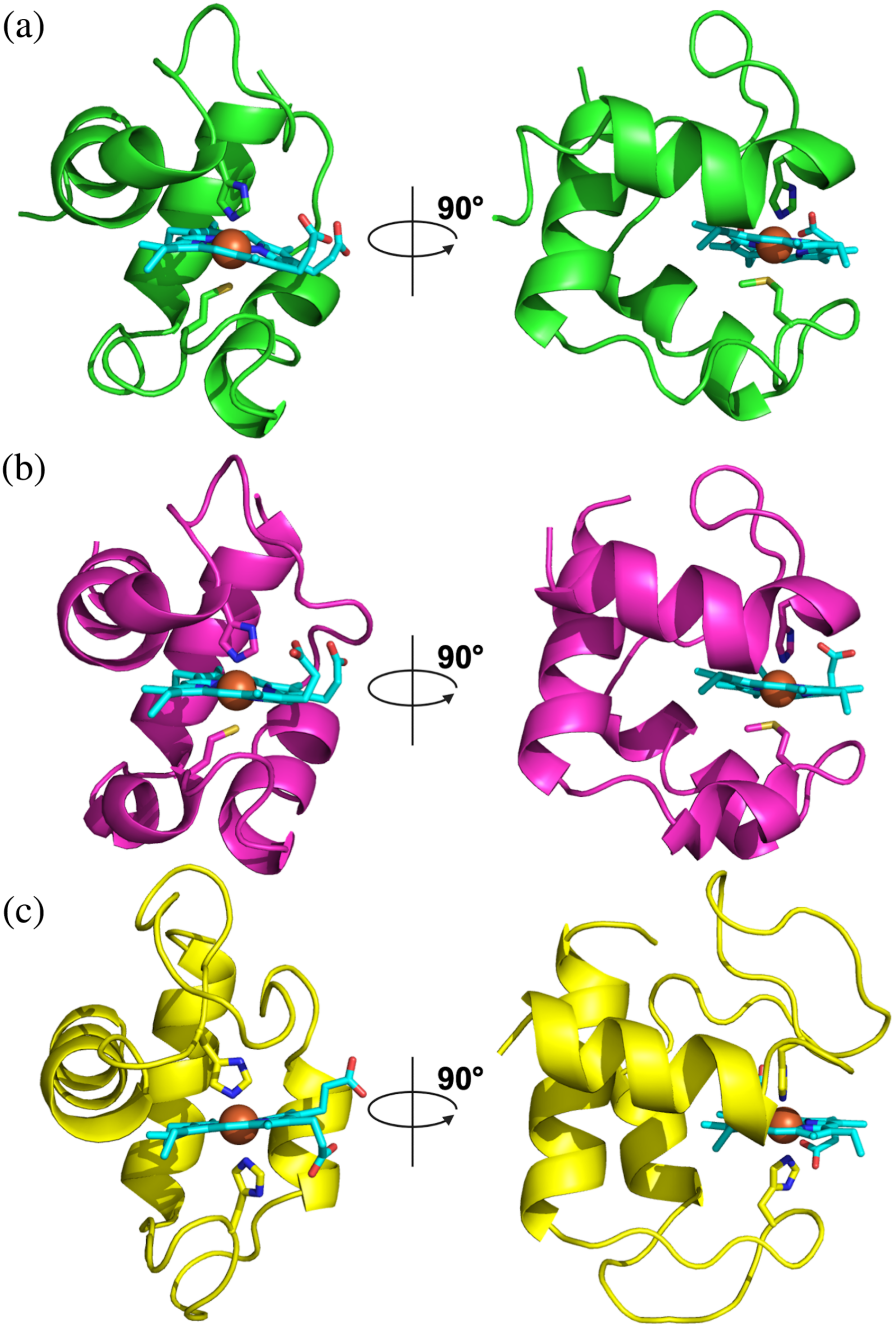
X‐ray crystal structures of PgcA monoheme domains. Front and side views are displayed in cartoon format. (a) Monoheme domain I, green, at 1.55 Å resolution (PDB ID: 8QJ6). (b) Monoheme domain II, magenta, at 1.80 Å resolution (PDB ID: 8QJG). (c) Monoheme domain III, yellow, at 1.90 Å resolution (PDB ID: 8QK0). Heme and iron‐ligating residues (His274, Met301, His365, Met392, His459, and His491) represented with sticks.

The atomic structures of the three PgcA monoheme domains were resolved by X‐ray crystallography (Figure 2 and Table S1). The 1.55 Å crystal structure of monoheme domain I reveals a globular arrangement comprising residues 261–321 from the PgcA amino acid sequence (Figure 2a). The protein contains a single heme cofactor covalently attached via thioether bonds to the cysteines of the CXXCH motif. The heme is enclosed in a peptide fold consisting of three short α‐helices separated by flexible loops, with the CXXCH motif located within the first α‐helix. The axial ligation of the heme iron is provided by His274 on the proximal side and Met301 on the distal side. The imidazole group of His274 locates within H‐bonding distance (2.8 Å) of the carboxyl group of Glu281 (Figure S3A). This interaction could influence the properties of the heme group, maintaining a suitable E_m_ to facilitate the transfer of electrons onto low‐potential minerals.

The 1.8 Å crystal structure of heme domain II consisted of six copies of the protein within the asymmetric unit, comprising residues 352–414 of the PgcA amino acid sequence. The overall fold is the same within each copy and consists of four loop‐linked α‐helices encapsulating the *c*‐type heme moiety (Figure 2b). The structures of domains I and II are highly similar, with a root mean square deviation (RMSD) of 0.453 Å and share 53% sequence identity. Like domain I, domain II displays His‐Met heme ligation (in this case from residues His365 and Met392) and the imidazole groups of His365 are within H‐bonding distance (2.7 Å) of the carboxylate group of Asp372 (Figure S3b). This suggests that PgcA domains I and II likely arose from a gene duplication event and that both the heme axial ligands and the histidine coordinated acidic residues are important structural features of these domains.

The 1.9 Å crystal structure of heme domain III was composed of PgcA residues 444–511 and is significantly different from those of domains I and II (Figure 2c). First, the coordination of the iron atom within the *c‐*type heme is bis‐His (His459 and His491), as commonly observed in multiheme cytochromes. Second, no acidic side chain is observed near the distal ligating histidine residue. Domain III contains only three α‐helices despite its molecular weight being ~1 kDa larger than those of the preceding two domains (Figures S1b and S2). In fact, a greater fraction of its polypeptide chain forms loops that likely possess greater flexibility when unrestricted by crystallization. This crystal structure also shows that 33% of the total surface area of the *c*‐type heme moiety is solvent exposed, which is substantially larger than the 25% exposure of the hemes in domains I and II.

### 2.3 Spectroscopic and potentiometric characterization of PgcA domains

The *E*_m_ values of the PgcA monoheme domains were previously measured by spectropotentiometric titration and were between −48 and −106 mV versus the standard hydrogen electrode (SHE) (Fernandes et al., 2023). In this study, protein film voltammetry (PFV) was used to determine the effect of pH on the monoheme domains and probe the redox properties of triheme PgcA. The adsorbed monoheme domain proteins yielded single reversible redox peaks on the indium tin‐oxide (ITO) electrodes across a pH range of 5–8 (Figure S4) consistent with previous measurements (Fernandes et al., 2023) (Table S2). Domains I and II specifically displayed much greater pH dependence, while domain III only shifted by ~10 mV, which is surprising considering the higher solvent accessibility of the domain III heme. The explanation likely lies in differences in amino acid side chains within the environment of the hemes, possibly the role played by Glu281 and Asp372.

**FIGURE 3:**
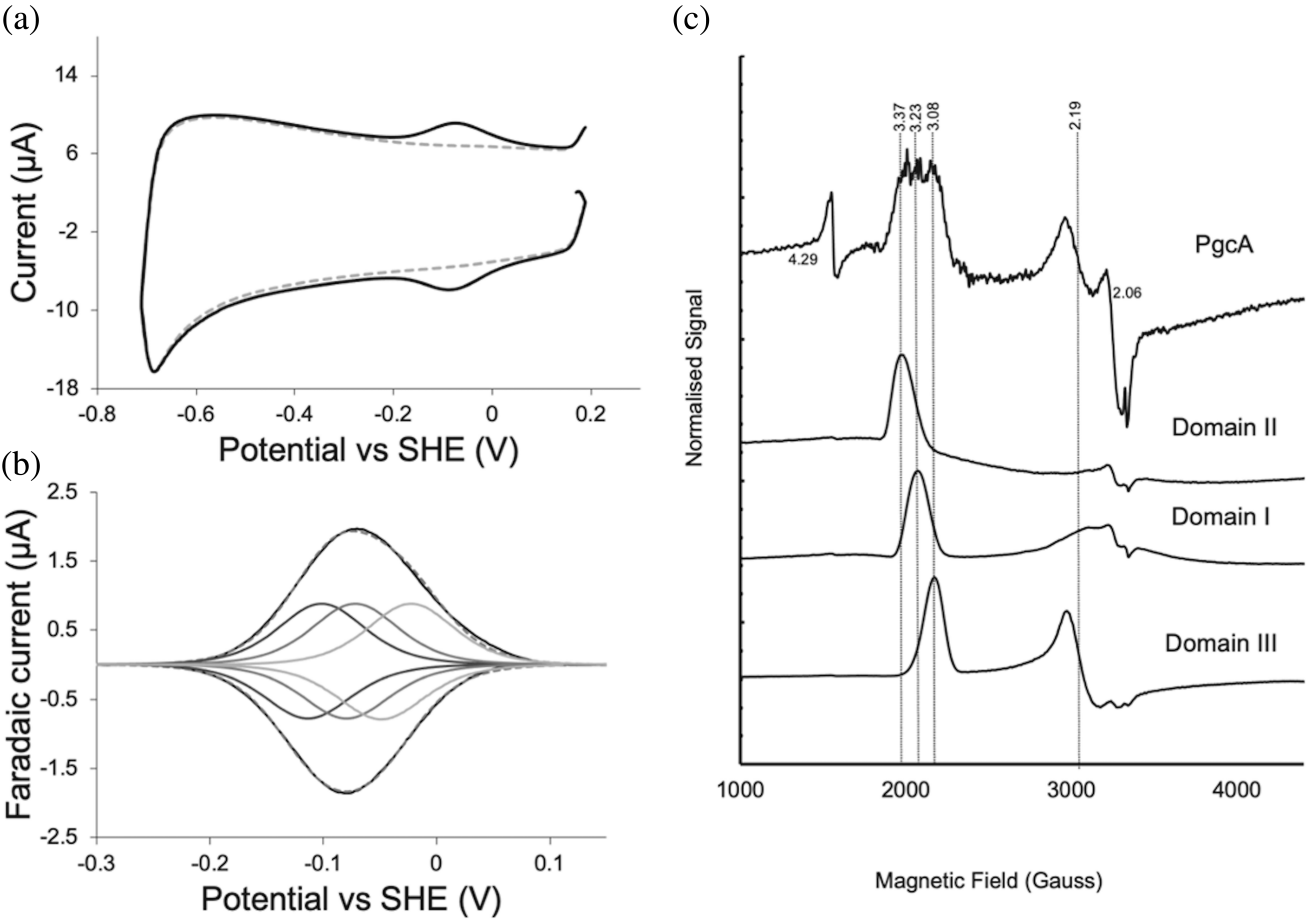
Voltametric and EPR spectroscopic properties of triheme PgcA. (a) Representative 0.01 V s^−1^ protein film voltammogram of triheme PgcA adsorbed on an ITO electrode in 50 mM Tris–HCl, 50 mM NaCl pH 8.0 (black), and untreated electrode (gray dotted). (b) Baseline subtracted voltammogram (0.01 V s^−1^) of triheme PgcA experimental data (black), three modeled *n* = 1 centres (gray), and their sum (gray dotted). (c) 10 k EPR spectra of triheme PgcA and its monoheme domains. The *g*‐values of individual EPR lines are indicated by vertical lines. The low‐field g1 components of the LS ferric heme in the three domains perfectly fit their contributions in the triheme PgcA spectrum. The signals at *g* = 4.29, *g* = 2.06, and *g* = 2.00 are likely from adventitious non‐heme Fe^3+^, Cu^2+^, and small free radicals, respectively.

Adsorption of triheme PgcA onto optically transparent ITO electrodes yielded UV–visible absorbance spectra with a shift in absorption maxima from 408 to 419 nm as the working electrode potential was lowered (Figure S5), consistent with the full reduction of PgcA on an electrode. Cyclic voltammetry of triheme PgcA displayed reversible reduction between −200 and +100 mV of approximately equal area for reductive and oxidative scans (Figure 3a), and fitting of this data to three equal contributions from individual *n* = 1 centers suggested three *E*_m_ values of approximately −108, −76 and −35 mV at pH 8.0, close to the respective individual *E*_m_ values for the individual domains (Figure 3b).

Figure 3c shows the electron paramagnetic resonance (EPR) spectra of the triheme PgcA protein and its individual isolated domains, I–III. Each individual domain has a clear low spin (LS), *S* = ½ EPR signature and, when normalized, represent equal contributions to the triheme PgcA spectrum. The domain III spectrum is a small *g*_max_, rhombic type LS signal (*g*_max_ 3.08) typical of bis‐histidine coordinated hemes with near parallel ligand orientation. From domain III to domain I to domain II, the spectra have increasingly anisotropic signatures where the *g*_max_ value becomes more intense than the other two *g*‐values (De & Albracht, 1979) (*g*_max_ = 3.23 and 3.37 for domain I and II, respectively). A highly anisotropic or axial LS (HALS) type “large *g*_max_” signal (*g* > 3.2) can occur when two histidine heme ligands are perpendicular to each other but is also typical of histidine–methionine heme coordination where ligand orientation cannot be resolved (Zoppellaro et al., 2009).

### 2.4 Biophysical analysis of the solution structure of PgcA

**FIGURE 4:**
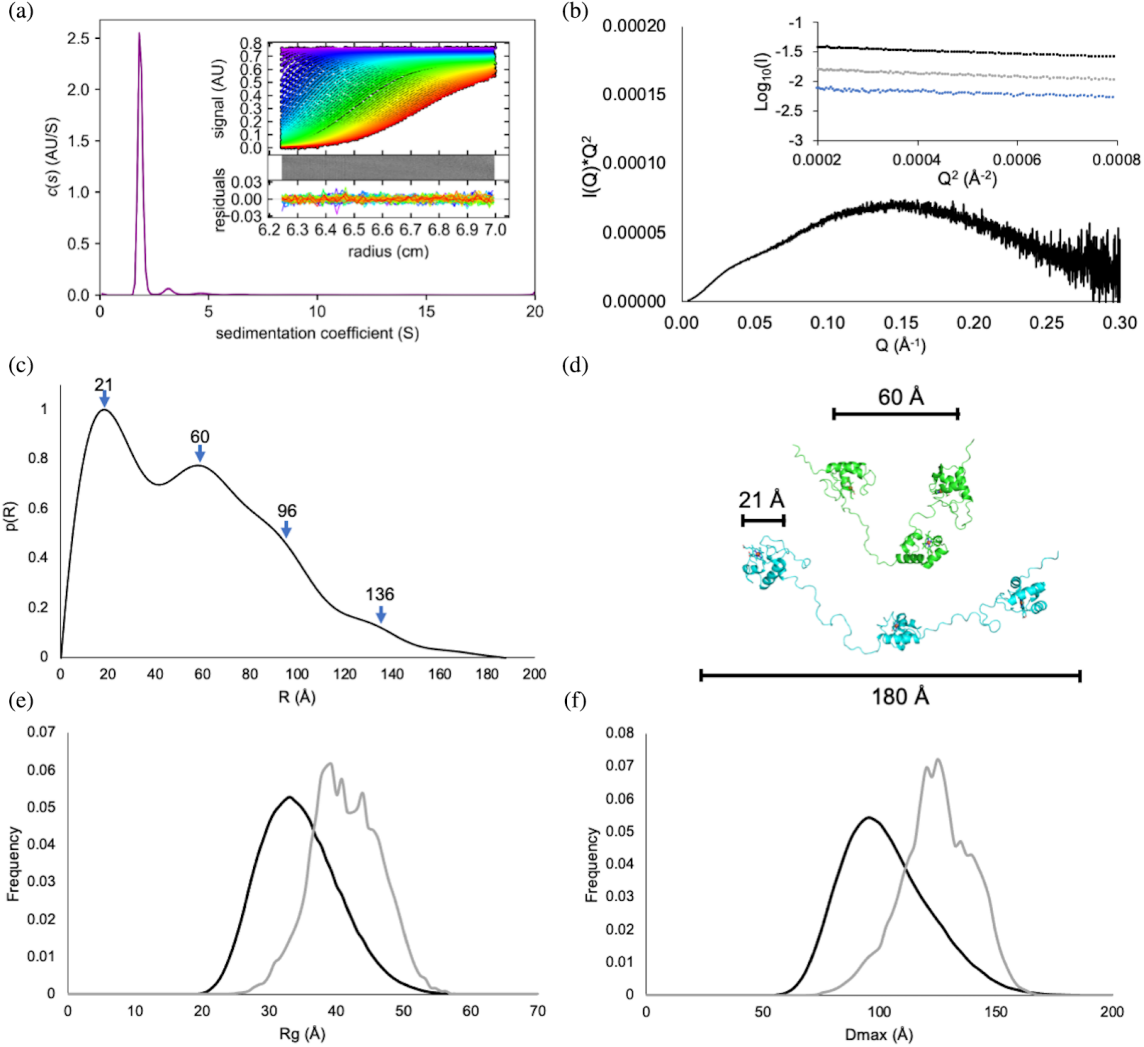
Biophysical solution state analysis of triheme PgcA. (a) c(S) distribution for 2 μM triheme PgcA in 100 mM Tris–HCl 100 mM NaCl pH 8.0, at 20°C. Inset top: raw data sedimentation velocity and fit. Inset middle: 2D difference map. Inset bottom: residual difference plot. (b) SEC‐SAXS Kratky plot and Guinier approximation plot for triheme PgcA at 10 (black), 5 (gray), and 2.5 (blue) mg mL^−1^ in 50 mM Tris–HCl 50 mM NaCl pH 8.0. (c) p(R) distance distribution modeled for triheme PgcA. (d) Two representative PgcA structural conformation models from the three‐state model, generated by MultiFoXS fitting to experimental scattering. (e,f) Frequency distribution plots generated by EOM analysis of triheme PgcA SEC‐SAXS scattering curve. Initial randomized ensemble shown in black, optimized ensemble with improved fit to the experimental data in gray, Results are the average of four EOM outputs.

The biophysical properties of PgcA and the three monoheme domains were analyzed using sedimentation velocity (Figure 4a and Figure S6). Continuous sedimentation distribution c(S) analysis revealed single, monodisperse species with *S*_20,W_ values of 1.17, 1.27, 1.36 S, corresponding to molecular weights of 8.9, 9.5, and 10.5 kDa for domains I, II, and III, respectively (Figure S6a–c). c(S) analysis of a mixed sample containing all three domains revealed a single peak with an *S*_20,W_ value of 1.28 S, indicating no significant complex formation between the three domains (Figure S6d). c(S) analysis of the triheme PgcA revealed a major peak at 1.98 S (31.2 kDa), consistent with the presence of a single PgcA species in solution (Figure 4a). The fitted *f*/*f*_0_ ratio of this species was 1.81, indicating a significantly elongated structure rather than the globular structures of the monoheme domains (*f*/*f*_0_ ~ 1.3). The data are consistent with three heme‐containing domains being connected by flexible linkers rather than a closely packed globular structure.

Size‐exclusion chromatography coupled with small‐angle x‐ray scattering (SEC‐SAXS) was undertaken on triheme PgcA samples at 2.5–10 mg mL^−1^. Samples eluted at the same positions and gave a uniform radius of gyration (*R*_g_) at higher scattering intensities (Figure S7a). Scans in this region were averaged and compared to the median to generate appropriate scattering curves (Figure S7b). Guinier analysis of the scattering curves at each concentration remained constant to low *Q* and gave *R*_g_ values of 39 ± 0.4, 44 ± 0.3, and 43 ± 0.2 for 2.5, 5, and 10 mg mL^−1^ concentrations, respectively (Figure 4b, inset). Taken together, these results indicate that PgcA remains monodisperse across the experimental concentration range. Therefore, the sample at 10 mg mL^−1^ was used for further analysis. Plotting the data as a function of *I*(*q*) × *q* ^2^ versus *q* (Kratky plot, Figure 4b) yielded a scattering curve with a stretched, broad shoulder displaying maxima at *q* = 0.15 Å^−1^, rather than the typical sharp, near‐Gaussian peak at low *q* values associated with folded proteins. This is consistent with the hypothesis that the triheme PgcA structure contains disordered regions.

The probability distance distribution *p*(*r*) curve for the PgcA scattering profile yielded a broad curve with identifiable peaks at 21, 60, 96, and 136 Å, before reaching a *D*_max_ of 180 Å (Figure 4c). This profile provides vital information about the dynamics of PgcA in solution. The peak at 20 Å results from the interatomic distances within each of the three domains, the peak at 60 Å likely represents the average interatomic distances between adjacent domains I and II or II and III, and most importantly, the remaining two peaks likely result from the average interatomic distances between domains I and III, which exist in a range of conformations and stretch as far apart as 180 Å. Using the modeling program MULTIFOXS (Schneidman‐Duhovny et al., 2016), the experimental scattering data was fitted to a range of models generated from the crystal structures of the three cytochrome domains connected with flexible (PT)_14_ linkers (Figure 4d and Figure S7c). The minimum number of models required to fit the experimental data was two, representing different averaged conformations of PgcA in solution (Figure S7d). Further analysis using the Ensemble optimization method (EOM), consistently revealed that most conformations adopted by PgcA exist between *R*_g_ values of 30 and 55 Å and between Dmax values of 70 and 160 Å, suggesting that intermediate extension of the protein dominates over the rarer contracted and highly extended forms.

### 2.5 Interactions of triheme PgcA and PgcA monoheme domains with iron(III) oxides

The affinity of proteins for insoluble iron(III) oxides can be measured using pull‐down assays where proteins are incubated in a suspension of iron(III) oxides before centrifugation. The concentration of protein remaining in solution is measured spectroscopically, allowing determination of the binding extent. Using this method, the interaction of PgcA and monoheme domains with the iron(III) oxide akageneite was measured. We observed that triheme PgcA and domain III bind to akageneite with similar affinity (Figure S8), while neither domains I or II bind at all. These results were consistent with PgcA interacting with minerals through domain III only, suggesting the flexible (PT)_x_ linkers which were previously proposed (Fernandes et al., 2023; Zacharoff et al., 2017) to increase the affinity of PgcA for iron(III) oxides, make a negligible contribution to binding. The selectivity of domain III for binding to akageneite is striking given that all three domains bind with high affinity to the mesoporous ITO electrodes used for PFV.

**FIGURE 5:**
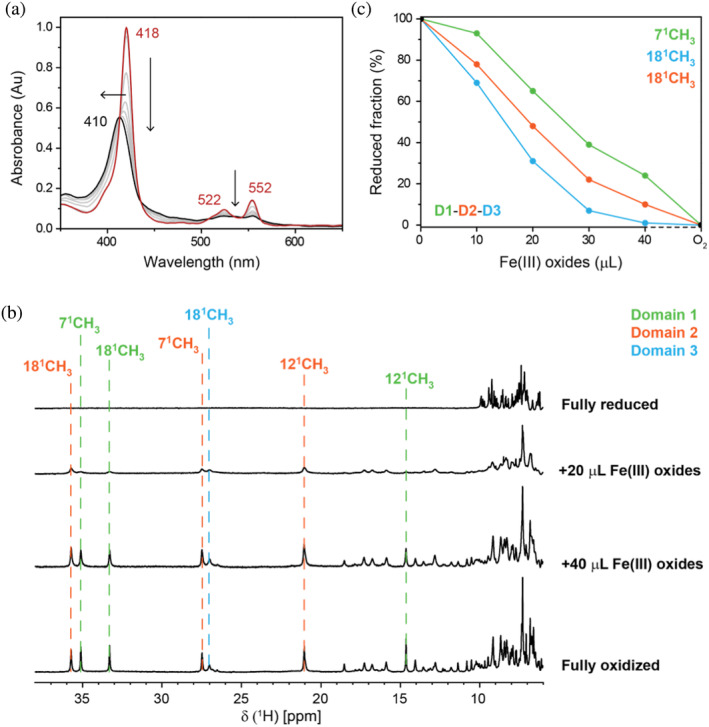
Reduction of iron(III) oxides by triheme PgcA. (a) UV–visible spectra of triheme PgcA across iron(III) oxides titration (black and gray). The fully reduced cytochrome is represented in red. (b) Variation of the low‐field 1D ^1^H NMR spectra of triheme PgcA across iron(III) oxides titration. Dashed lines indicate the different heme methyl resonances of the monoheme domains I (green), II (orange), and III (blue). (c) Reduced fractions of each monoheme domain across iron(III) oxides titration, deconvoluted from 1D ^1^H NMR spectra.

UV–visible spectroscopy was used to monitor the electron transfer reaction between triheme PgcA and iron(III) oxides. Titration with iron(III) oxides caused the complete oxidation of all three hemes within the sample (Figure 5a). This is notable, as previously only domain III was shown to reduce iron(III) oxides (Fernandes et al., 2023), suggesting that domains I and II oxidize solely via electron transfer onto domain III.

The UV–visible spectral features of the three cytochrome domains are not easily distinguished, but their 1D ^1^H‐NMR spectra present unique and identifiable signal patterns (Fernandes et al., 2023). The 1D ^1^H‐NMR spectra of the triheme PgcA in the fully reduced and fully oxidized states are well resolved (Figure S9), and the resonances superpose well with those of the individual monoheme domains (Fernandes et al., 2023). We exploited this to track the individual redox states of each domain in the triheme PgcA following sequential additions of iron(III) oxides (Figure 5b). The reduced fractions of each domain decreased along the titration, according to their *E*_m_, following the expected thermodynamic route. While these experiments cannot distinguish between inter‐ and intra‐PgcA electron transfer pathways, these observations show clearly that domain III directly reduces iron(III) oxides, while domains I and II are able to recharge domain III for further electron transfer.

## 3 DISCUSSION

The transfer of electrons through chains of redox‐active cofactors is a fundamental process, ubiquitous in all organisms. To ensure that electron transfer is both rapid and controlled, cofactors must be packed together so the overall distance between edges is typically <15 Å (Moser et al., 1992). For *c*‐type cytochromes, these edge‐edge distances are often <5 Å, allowing for electron transfer on a nanosecond timescale across multiheme proteins (Breuer et al., 2015; Clarke et al., 2011; van Wonderen et al., 2021; Wang et al., 2019). These heme wires can be over 120 Å in the periplasm or 180 Å across the outer membrane, while polymers of *c*‐type cytochromes, such as OmcS, OmcZ, and OmcE in *G. sulfurreducens*, can reach micrometers in length (Wang et al., 2019; Wang, Chan, et al., 2022; Wang, Mustafa, et al., 2022). These wires allow for rapid electron transfer between electron donors and terminal electron acceptors but require the assembly of multiple heme groups that are costly to synthesize.

In this report, we reveal the properties of a new type of multiheme cytochrome that consists of individual *c*‐type cytochrome domains tethered together by (PT)_x_ linker peptides. Structural and spectroscopic analysis of the three domains show them to be distinct, highly solvent‐exposed monoheme proteins, two of which have His–Met heme coordination and one that is His–His coordinated (Reedy et al., 2008). Domains I and II possess two acidic residues, Glu281 and Asp372, which hydrogen bond the proximal histidine of the His–Met coordinated heme. These negatively charged residues might help stabilize the Fe^3+^ state over Fe^2+^, supported by the pH‐dependent voltammograms which show that these two domains have *E*_m_ that is significantly more pH sensitive than domain III.

The multimodal SAXS p(R) curve of the triheme PgcA is consistent with three freely moving, but tethered, domains. The maximum at 20 Å represents the distances between atoms located in the same domain, while the maximum at 60 Å represents the distances between atoms in different domains, at the most prevalent interdomain distance. This explains the exceptionally high radius of gyration and implies that the shallow *p*(*r*) decrease to a *D*_max_ of >180 Å is derived from a small population of the most extended conformations of the chain. The *D*_max_ of 180 Å therefore corresponds to the greatest interatomic distance within the most extended conformer(s). The fact that the NMR spectrum of the triheme protein is the sum of the spectra of the three domains, and that the signals have similar narrow linewidths to those observed for the individual domains, also indicates that the domains tumble freely and independently in solution.

Sequence alignments of the (PT)_x_ motifs from PgcA and its homologues have shown them to be highly conserved (Zacharoff et al., 2017), and for many years, the motif has been proposed to facilitate interactions with minerals (Lower et al., 2008). Our findings require that this hypothesis is revised. We do not detect differences in the binding affinity for the triheme PgcA and domain III with iron(III) oxide. Thus, we find no evidence for the role of (PT)x motifs in this interaction. Why then is the (PT)_x_ motif conserved? Proline is a torsion‐constrained amino acid that prevents the formation of secondary structure, so its incorporation must heavily impact the flexibility of the linker regions. When the PgcA sequence is analyzed by ExPASy‐*PeptideCutter* (Wilkins et al., 1999), only proteinase K can cleave the (PT)_x_ motif. This suggests this sequence would be resistant to cleavage by many proteases, a feature essential for a protein exported into the extracellular environment. These observations suggest that the (PT)_x_ motifs serve as linkers that maintain an optimal distribution of distances between cytochrome domains, allowing for electron transfer to occur between them, while ensuring the ability to stretch across 18 nm.

The interaction studies indicate that only domain III, with the most solvent exposed heme, is capable of interacting with iron(III) oxides under our experimental conditions. While these are unlikely to represent the physiological and environmental conditions under which PgcA is active, they provide a useful mechanism for studying interdomain electron transfer between the different domains. Our results clearly show that all three domains of triheme PgcA become oxidized by iron(III) oxides, indicating that electrons are likely transferred from domains I–II onto domain III, and then onto minerals, as neither domains I nor II are oxidized alone by iron(III) oxides (Fernandes et al., 2023). This was confirmed by the NMR studies, which revealed that domain III was the first to be oxidized upon exposure to iron(III) oxides, followed by domains II and I.

The novel flexible‐movement‐based structural system we describe maximizes the chance of mineral‐PgcA contact while allowing electron transfer between the domains. The rate of electron transfer is likely to be orders of magnitude slower than those of closely‐packed heme wire proteins (van Wonderen et al., 2021), so PgcA probably performs a complementary function to those proteins, rather than a parallel one. For example, in instances where mineral–cell contact occurs, transport of electrons across the outer membrane will be rapid exclusively at the point of mineral contact. A large number of PgcA proteins might facilitate rapid lateral electron transfer across the cell's outer surface, allowing porin–cytochrome complexes not in direct contact with the mineral to indirectly reduce it.

Using the known structures, mass, and heme number of cytochrome wires, we can predict that for a similar wire to span the 18 nm we measured for PgcA, it would require a 24‐heme arrangement with a mass of 85–125 kDa. This mechanism for long range electron transfer on minimal heme chassis is ubiquitous, as genes encoding extracellular PgcA paralogs containing 3–14 hemes have been identified in the genomes of disparate bacteria, such as *Geotalea luticola*, *Citrifermentans bemidjiensis*, and *Pelobacter selenigens* (Fernandes et al., 2023). The specific roles of these genes are not yet defined, but the information provided by this work suggests that long‐range extracellular electron transfer through tethered hemes is potentially an important respiratory strategy for environmental microorganisms.

## 4 METHODS

### 4.1 Plasmid design and construction

The PgcA gene was provided by Prof. Daniel Bond (University of Minnesota) in pBAD202PgcA and transformed into *S. oneidensis MR‐1* strain LS527. Plasmid DNA was purified, and PCR mutagenesis was performed to generate first the pBAD202PgcA‐strep construct, from which the three single domain constructs were subsequently generated in one and two rounds of mutagenesis for domain III and domains I and II, respectively. The linear blunt ended DNA fragments were circularized with T4 kinase and ligase before heat shock transformation into *E. coli* TOP10 and subsequent electroporation back into *MR‐1*. Primers and respective plasmids are shown in Table S3.

### 4.2 Expression and purification of PgcA and its constituent domains

Colonies were transferred into 100 mL LB‐media (30 μg mL^−1^ kanamycin) and grown aerobically overnight, before 1.5% inoculation of 1 L LB in baffled flasks. These were grown until OD_600_ ≈ 0.5 was reached, 3 mM arabinose was then added after which they were incubated overnight, all growth at 30°C 180 RPM. Cells were harvested by centrifugation and lysed by double pass through a French press at 1000 PSI. Cell lysate was clarified by ultracentrifugation, the soluble fraction was then loaded onto a Strep‐Tactin® XT 4Flow® column equilibrated with 100 mM Tris–HCl, 150 mM NaCl pH 8.0, washed, and eluted with 50 mM biotin according to the manufacturer's guidelines. Triheme PgcA and the monoheme domains were then concentrated using 3 kDa MWCO spin concentrators and further purified by SEC through a cytiva HiLoad 16/600 Superdex 75 pg. column into 20 mM HEPES, 100 mM NaCl pH 7.8. Purity was assessed throughout using SDS‐PAGE stained with Coomassie and peroxidase linked heme stain (Thomas et al., 1976), and 410–280 nm absorbance ratio. Molecular weight of purified proteins was determined by LC–MS as described in ref (van Wonderen et al., 2018). Protein anticipated molecular weights calculated by inputting the amino acid sequence to ExPASy ProtParam (Wilkins et al., 1999) and including a mass of 615.17 Da per heme.

### 4.3 Small‐angle x‐ray scattering (SAXS) and analytical ultracentrifugation

Triheme PgcA in 50 mM Tris 50 mM NaCl pH 8.0 was concentrated to 10, 5, and 2.5 mg mL^−1^ and analyzed on B21 at Diamond light source. Samples were loaded onto a Shodex KW‐402.5 SEC column equilibrated in the same buffer and data was recorded using an Eiger 4 M detector. Data were analyzed using ScÅtter IV (bl1231.als.lbl.gov/scatter/) where scans with uniform radius of gyration (*R*_g_) values were averaged, and baseline was subtracted using pre‐protein peak scans (Figure S7). Guinier, Kratky and plots were generated with Primus (Manalastas‐Cantos et al., 2021). The Primus distance distribution wizard Autognom function was then used to generate a *p*(*R*) plot (Manalastas‐Cantos et al., 2021). The 10 mg mL^−1^ scattering data and triheme PgcA sequence were uploaded to the MultiFoxS and EOM servers and modeled with default parameters using the crystal structures specified as rigid bodies and the (PT)_X_ sequences as flexible linkers (Tria et al., 2015). Sedimentation velocity samples were diluted to 2 μM in 100 mM Tris–HCl, 100 mM NaCl, pH 8. Samples were centrifuged at 129,000 G, 20°C, and migration through the cell monitored at 408 nm. Scans were analyzed using the software program Sedfit in c(S) distribution mode (Schuck, 2000).

### 4.4 Crystallization and structure determination of PgcA monoheme domains

Crystals of heme‐domain I were obtained by sitting‐drop vapor diffusion by depositing a droplet containing 0.3 μL 10 mg mL^−1^ protein in 20 mM HEPES pH 7.8, 100 mM NaCl, onto a 0.28 μL drop of reservoir solution containing 2.5 M Na malonate pH 7.0 and 0.02 μL 30% w/v PEG 2000 MME at 16°C. Crystals were cryoprotected by transferring to 20% ethylene glycol 2.5 M Na malonate pH 7.0 before vitrification.

Crystals of heme‐domain II were obtained by sitting‐drop vapor diffusion using a 1:1 mixture of 10 mg mL^−1^ protein in 20 mM HEPES pH 7.8, 100 mM NaCl and 2.0 M ammonium sulfate, 5% v/v isopropanol, and 1.8% w/v PEG 4000 at 16°C with a total drop volume of 0.6 μL. Crystals were cryoprotected by transferring to 20% ethylene glycol, 2.0 M ammonium sulfate, and 5% isopropanol before vitrification.

Crystals of heme‐domain III were obtained by sitting‐drop vapor diffusion using a 1:1 mixture of 20 mg mL^−1^ protein in 20 mM HEPES pH 7.8, 100 mM NaCl, and 2.3 M Na malonate pH 7.0, 1.8% Jeffamine ED‐2001 at 16°C with a total drop volume of 0.6 μL. Crystals were cryoprotected by transferring to 25% glycerol and 2.4 M Na malonate, pH 7, before vitrification.

Datasets were collected on frozen crystals at Diamond beamline I04 and I24 with x‐rays of wavelength 0.9537 Å. Datasets were indexed and scaled automatically with XIA2 (Winter, 2010) and merged with AIMLESS (Evans & Murshudov, 2013). Initial models were generated by molecular replacement with AF2 (Jumper et al., 2021) models in PHASER (McCoy et al., 2007) or MOLREP (Vagin & Teplyakov, 1997); these models were refined manually in COOT (Emsley et al., 2010) using PHENIX.REFINE (Liebschner et al., 2019) and REFMAC5 (Murshudov et al., 2011) to obtain final coordinates. Data collection and model refinement statistics are listed in Table S1.

### 4.5 Protein film voltammetry

Hierarchical mesoporous ITO electrodes were prepared as described previously (Mersch et al., 2015). 10 mg mL^−1^ Protein samples in 20 mM HEPES pH 7.8, 100 mM NaCl were deposited onto ice cold ITO electrodes and incubated for ~5 min. After rinsing, electrodes were introduced to anaerobic buffer‐electrolyte solution within a glass electrochemical cell, inside a Faraday cage and situated in a N_2_‐filled chamber (atmospheric O_2_ < 2 ppm). Cyclic voltammetry was performed with a three‐electrode configuration using an Autolab PGSTAT30 potentiostat (EcoChemie) controlled by NOVA software. An Ag/AgCl (saturated KCl) reference electrode was used with a Pt wire counter electrode, and potential values corrected to SHE by addition of +0.197 V. Charging current baselines were subtracted from the measured voltammograms to yield the Faradaic currents. For monoheme domains Faradaic currents were fit to single *n* = 1 Nernstian peaks using QSoas (Fourmond, 2016). Peak potentials for the oxidative and reductive signals were averaged across three independent scans, and standard error calculated, to obtain the reported *E*_m_ values. For triheme PgcA, Faradaic currents were fit to three Nernstian *n* = 1 centers of equal area using QSoas (Fourmond, 2016). For optically monitored potentiometric titration of ITO coated electrodes, those electrodes were studied in anaerobic buffer‐electrolyte solution within a sealed cuvette fitted with a Pt wire as counter electrode and an AgCl‐coated Ag wire as a reference electrode. The cuvette was placed in a UV–visible absorbance spectrophotometer and the working electrode poised at the desired potentials. The reference electrode was calibrated by cyclic voltammetry with potassium hexa‐cyanoferrate(III) (30 mM in 50 mM buffer, 100 mM NaCl pH 8.0) taking *E*_m_ = +420 mV versus SHE.

### 4.6 EPR

EPR spectra were recorded at 10 K using a Bruker E500 (X‐band) EPR spectrometer and Oxford Instruments liquid helium systems. Instrument parameters were as follows: microwave frequency *ν*_MW_ = 9.467 GHz, modulation frequency *ν*_M_ = 100 kHz, time constant *τ* = 82 ms, microwave power = 3.19 mW, modulation amplitude *A*_M_ = 5 G, scan rate *ν* = 22.6 Gs^−1^. Wilmad SQ EPR tubes (Wilmad Glass, Buena, NJ) with OD = 4.05 ± 0.07 mm and ID = 3.12 ± 0.04 mm (mean ± range) were used for freezing EPR samples in methanol cooled with solid CO_2_. After freezing, the EPR tubes with samples were kept in liquid nitrogen until required for measurements.

### 4.7 Mineral oxide binding pull‐down assays

The binding of iron(III) oxides to triheme PgcA was probed by UV–visible spectroscopy, as described previously (Fernandes et al., 2023). Spectra were recorded between 300 and 700 nm using an Evolution 201 spectrophotometer (Thermo Scientific). After the acquisition of spectra with 2 μM protein samples prepared in 32 mM sodium phosphate buffer pH 7 with NaCl (100 mM final ionic strength), the protein was incubated with 55 mM of akageneite for 10 min, after which the resulting solutions were centrifuged at 5000*g* for 10 min. Additional spectra were acquired with the resulting supernatants and the putative binding of the protein to iron(III) oxides was evaluated based on differences in spectral intensity.

### 4.8 Iron(III) oxide‐mediated PgcA oxidation monitored by UV–visible spectroscopy

The electron transfer reaction between triheme PgcA and iron(III) oxides was assessed by UV–visible spectroscopy measurements at 25°C, performed inside an anaerobic glovebox with O_2_ levels kept under 0.1 ppm. Iron(III) oxides were prepared with a final concentration of 500 μM. The cytochrome samples (2 μM) were prepared in degassed 32 mM sodium phosphate buffer with NaCl (100 mM final ionic strength) at pH 7. Before each experiment, the protein was reduced with a solution of sodium dithionite. Iron(III) oxides were added to the fully reduced cytochrome in several increments and the consequent re‐oxidation of hemes was monitored by recording UV–visible spectra between 300 and 700 nm.

### 4.9 Iron(III) oxides reduction monitored by NMR spectroscopy

The electron transfer reaction between triheme PgcA and iron(III) oxides was also assessed by NMR spectroscopy. The samples of protein were prepared at 100 μM in 32 mM sodium phosphate buffer pH 7 (100 mM final ionic strength) in D_2_O and reduced with a solution of sodium dithionite. Iron(III) oxides were prepared with a final concentration of 18.75 mM in D_2_O. 1D ^1^H‐NMR spectra were acquired (i) in the reduced state, (ii) after 10 μL additions of iron(III) oxides solution, and (iii) after exposing the sample in the NMR tube to atmospheric O_2_. After each addition of iron(III) oxides, the samples were incubated for 5 min and centrifuged at 5000*g* for 10 min, prior to spectral acquisition. These experiments were acquired in a Bruker Avance III 600 MHz spectrometer. The residual H_2_O signal was used as an internal reference for the calibration of the ^1^H chemical shifts relative to sodium trimethylsilylpropanesulfonate (DSS) at 0 ppm. All spectra were acquired at 25°C and processed using TopSpin 4.1.4 (Bruker BioSpin, Karlsruhe Germany). The reduced fractions of each cytochrome domain along the redox reaction were calculated by integrating the ^1^H NMR heme methyl signals of each domain in the intermediate redox state, relative to the fully oxidized state.

## AUTHOR CONTRIBUTIONS

**Benjamin W. Nash:** Conceptualization; investigation; formal analysis; writing – original draft; writing – review and editing. **Tomás M. Fernandes:** Investigation; formal analysis; writing – review and editing. **Joshua A. J. Burton:** Investigation; writing – review and editing. **Leonor Morgado:** Formal analysis; writing – original draft; writing – review and editing. **Jessica H. van Wonderen:** Writing – review and editing; writing – original draft; investigation; formal analysis. **Dimitri A. Svistunenko:** Investigation; formal analysis; writing – review and editing; writing – original draft. **Marcus J. Edwards:** Formal analysis; writing – review and editing; investigation. **Carlos A. Salgueiro:** Formal analysis; funding acquisition; writing – original draft; writing – review and editing; supervision. **Julea N. Butt:** Formal analysis; writing – original draft; writing – review and editing; supervision. **Thomas A. Clarke:** Funding acquisition; conceptualization; writing – original draft; writing – review and editing; supervision; formal analysis.

## CONFLICT OF INTEREST STATEMENT

The authors declare no competing interests.

## Supporting information


**Data S1:** Supporting Information

## Data Availability

The data that support the findings of this study are openly available in PURE at https://research-portal.uea.ac.uk/en/datasets/nash-et-al-datasets-for-tethered-heme-domains-in-a-triheme-cytoch. Crystallographic coordinates and structure factors were deposited in the RCSB Protein Data Bank with PDB IDs 8QJ6 (Domain I), 8QJG (Domain II), and 8QK0 (Domain III).
