# Supplementary material for: Tethered heme domains in a triheme cytochrome allow for increased electron transport distances
Source: Protein Sci. 2024 Oct 29;33(11):e5200. doi: 10.1002/pro.5200 (PMC11520253; doi:10.1002/pro.5200)
Supplement: Supplementary file 1 — Data S1: Supporting Information [file PRO-33-e5200-s001.docx]

**Table S1: Data collection and refinement statistics for PgcA monoheme domain crystals.**

|  | Domain I (PDB 8QJ6) | Domain II (PDB 8QJG) | Domain III (PDB 8QK0) |
| --- | --- | --- | --- |
| **Data collection** |  |  |  |
| Space group | I 4 | C 1 2 1 | C 1 2 1 |
| Cell dimensions |  |  |  |
| *a*, *b*, *c* (Å) | 68.43, 68.43, 27.68 | 95.10, 74.10, 56.02 | 75.56, 55.03, 37.46 |
| α, β, γ (°) | 90.00, 90.00, 90.00 | 90.00, 103.92, 90.00 | 90.00, 99.30, 90.00 |
| Resolution (Å) | 24.19-1.55 (1.61-1.55) | 42.96-1.80 (1.86-1.80) | 44.28-1.9 (1.97-1.9) |
| *R*_pim_ (%)  *CC_1/2_* (%) | 2.0 (15.5)  99.9 (97.7) | 9.5 (26.5)  97.1 (84.5) | 5.7 (26.8)  99.6 (85.9) |
| *I* / σ*I* | 24.64 (2.90) | 7.93 (1.90) | 9.71 (1.64) |
| Completeness (%) | 97.12 (77.90) | 99.43 (95.91) | 99.57 (98.17) |
| Multiplicity | 12.9 (8.9) | 3.4 (2.6) | 6.8 (5.5) |
|  |  |  |  |
| **Refinement** |  |  |  |
| Resolution (Å) | 1.55 | 1.80 | 1.90 |
| No. reflections | 9226 | 34836 | 11995 |
| *R*_work_ / *R*_free_ | 0.155/0.189 | 0.183/0.231 | 0.221/0.263 |
| No. atoms |  |  |  |
| Protein | 439 | 2690 | 1028 |
| Ligand/ion | 44 | 327 | 96 |
| Water | 69 | 538 | 179 |
| *B*-factors |  |  |  |
| Protein | 14.86 | 13.83 | 18.12 |
| Ligand/ion | 12.69 | 14.40 | 19.46 |
| Water | 30.89 | 25.04 | 27.55 |
| R.m.s. deviations |  |  |  |
| Bond lengths (Å) | 0.009 | 0.010 | 0.008 |
| Bond angles (°) | 1.01 | 1.13 | 1.10 |

**Table S2: *E*_m_ values (mV vs SHE) and standard error, of PgcA monoheme domains determined by PFV**

| pH | E_m_ of domain I | E_m_ of domain II | E_m_ of domain III |
| --- | --- | --- | --- |
| 8 | -49 (±1) | -85 (±1) | -88 (±1) |
| 7 | -28 (±1) | -60 (±1) | -94 (±1) |
| 6 | -36 (±2) | -32 (±1) | -98 (±3) |
| 5 | -6 (±1) | -33 (±1) | -82 (±2) |

**Table S3: Primers and plasmids used in this study.**

| Plasmid | Product | Primers (5’ to 3’): |
| --- | --- | --- |
| pPgcA-strep | Triheme PgcA | Forward: GTTCGAAAAATGAGTTTAAACGGTCTCCCA  Reverse: TGCGGGTGGCTCCAACCACTACCGTTTGCGTTG |
| pPgcA-DI | Monoheme domain I | Pair 1 Forward: GGTAGTGGTTGGAGCCACCC  Pair 1 Reverse: AGCCACTGCCAAGACGTCAA  Pair 2 Forward:  GCCAACACAGGCTTGGCCGTCGCTGCTGATGGTGACGGACAAGGATTGTACGC  Pair 2 Reverse:  TAATAACGCTAGAGTGATCAAATTGAGTTTAAATTTCATCATGGGATGTATATCTC |
| pPgcA-DII | Monoheme domain II | Pair 1 Forward: GGTAGTGGTTGGAGCCACCC  Pair 1 Reverse: GACAGCCGATGCCAGCGAGG  Pair 2 Forward:  GCCAACACAGGCTTGGCCGTCGCTGCTGATGGTAACGGGTCGGCACTCTACGG  Pair 2 Reverse:  TAATAACGCTAGAGTGATCAAATTGAGTTTAAATTTCATCATGGGATGTATATCTC |
| pPgcA-DIII | Monoheme domain III | Forward: GCCAACACAGGCTTGGCCGTCGCTGCTGATGGTTAGTCGACCCGGGCAAGACCGT  Reverse: TAATAACGCTAGAGTGATCAAATTGAGTTTAAATTTCATCATGGGATGTATATCTCCTTCTTAGG |

**Figure S1: SDS-PAGE of PgcA fractions. (A)** Gel of PgcA fractions stained for heme. 1: Molecular weight markers. 2: PgcA fraction isolated by Strep-Tactin affinity chromatography. 3: ~ 30 kDa PgcA fraction isolated by size exclusion chromatography. **(B)** Coomassie stained gel of PgcA monoheme domains. 1: Molecular weight markers. 2: Domain I. 3: Domain II. 4: Domain III.

**
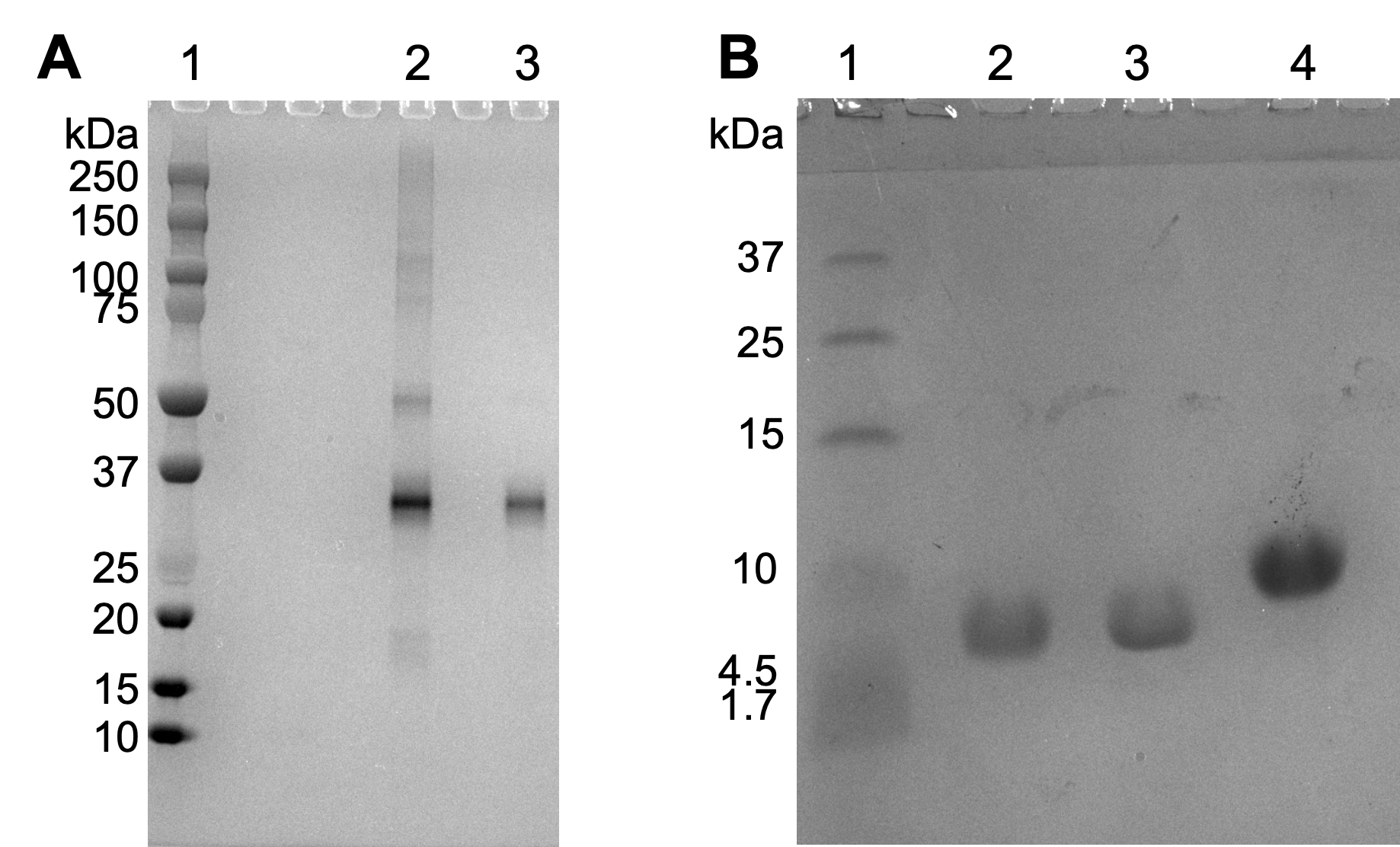
**

**Figure S2: LC-MS for monoheme domains.** Deconvoluted mass spectra for PgcA monoheme domains I (**A**), II (**B**) and III (**C**), Theoretical MWs calculated from mature sequence and 615.17 Da *c*-heme contribution are 8,152, 8,389 and 9,069 Da, respectively.

**
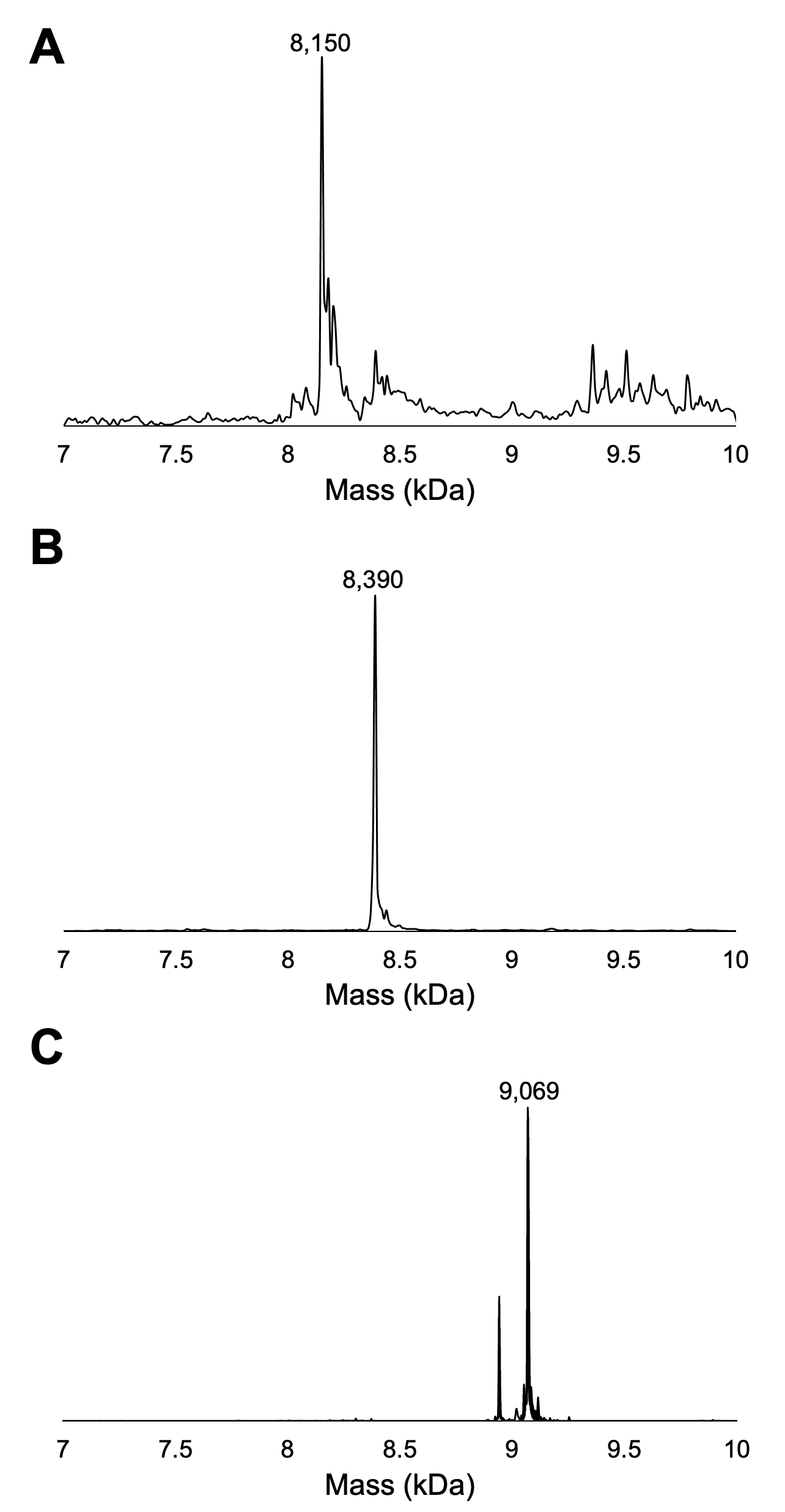
**

**Figure S3: Crystallographically resolved conformation of E281 and D372 sidechains.** Cartoon representation of PgcA monoheme domains I (**A**) and II (**B**) crystal structures at 1.55 and 1.8 Å resolution respectively, with heme iron-ligating residues (His274, Met301, His365 and Met392) and histidine H-bonding residues E281 and D372 represented with sticks.

**
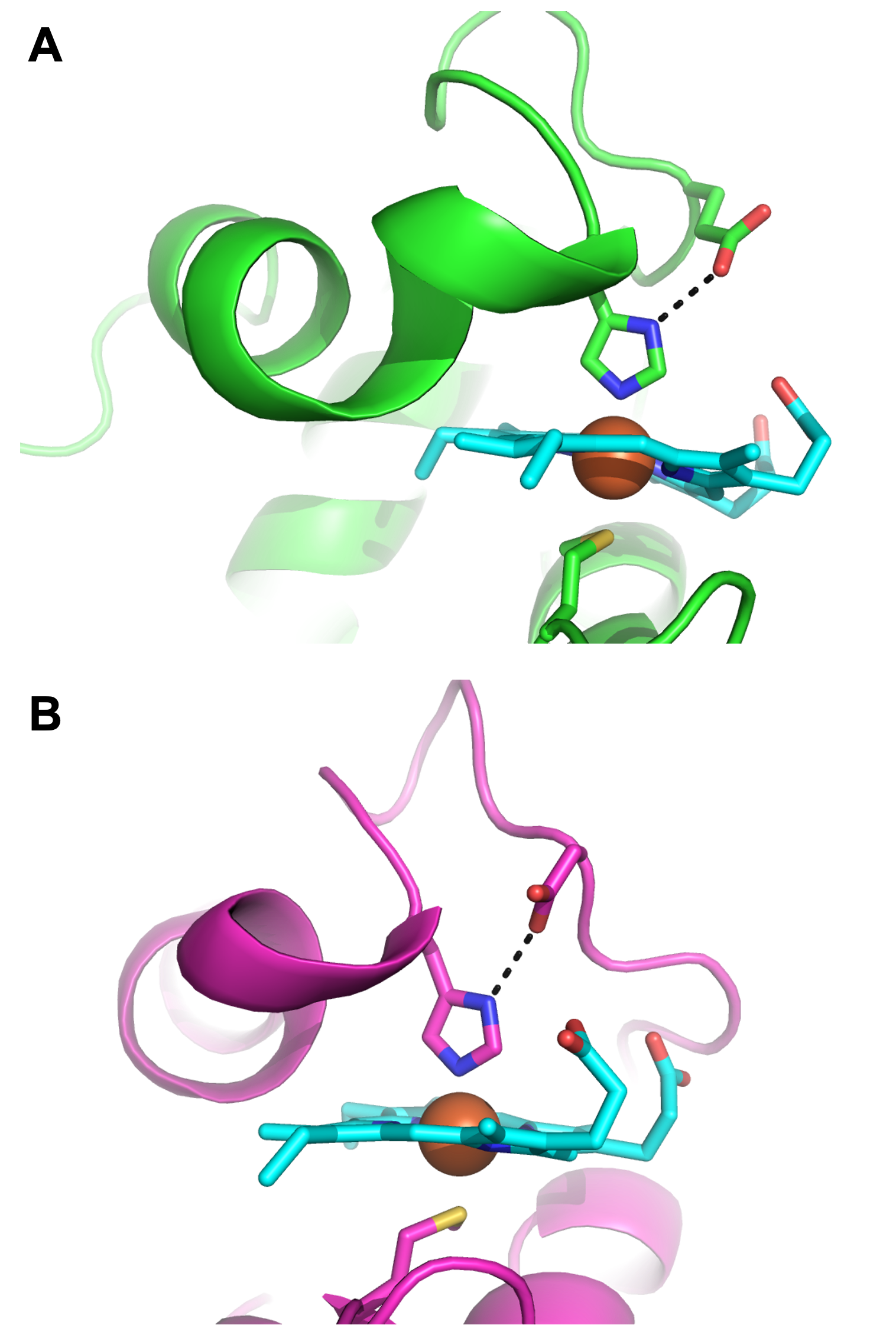
**

**Figure S4: Protein film voltammetry of monoheme domains.** Representative 0.01 V s^-1^ voltammograms of protein adsorbed onto ITO electrodes in 50 mM Tris-HCl, 50 mM NaCl pH 8.0 (grey continuous), untreated electrode (grey dotted) Faradaic currents (black). Data shown for domain I (**A**), domain II (**B**) and domain III (**C**).

**
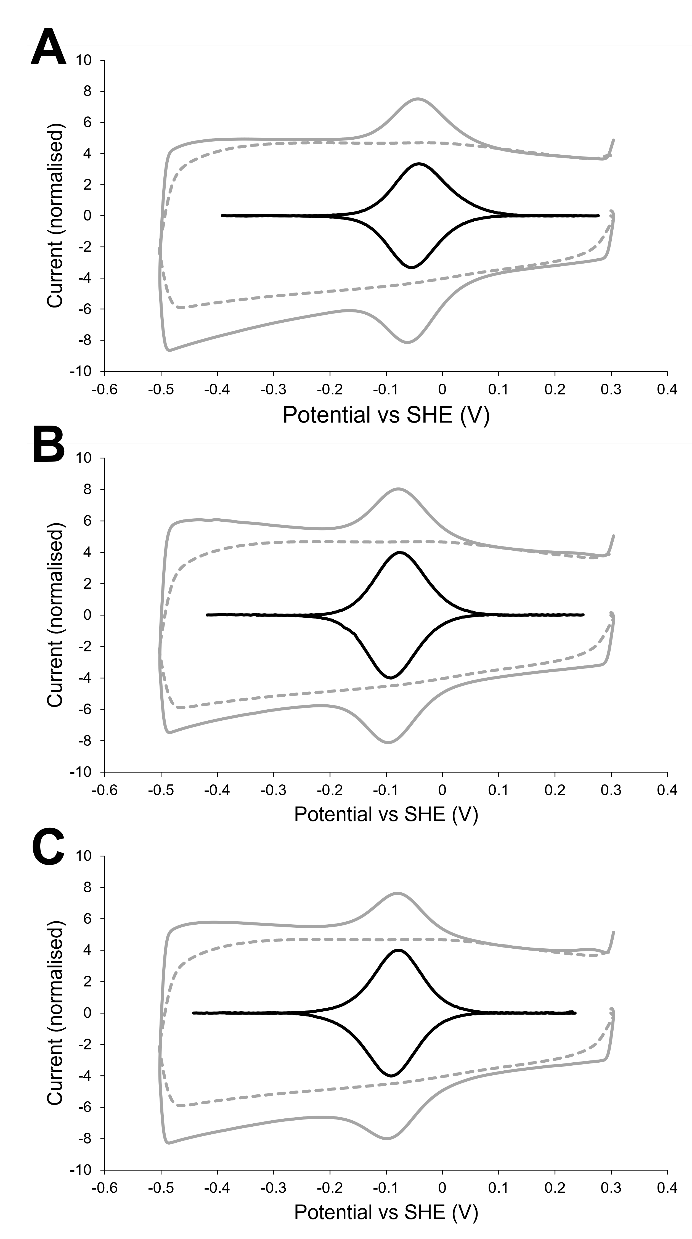
**

**Figure S5: UV-Visible spectra of triheme PgcA adsorbed onto ITO electrode.** Absorbance spectrum of triheme PgcA adsorbed onto mesoporous ITO electrode poised at -330 (grey) and +200 (black) mV vs SHE in 50 mM Tris-HCl, 50 mM NaCl pH 8.0.

**
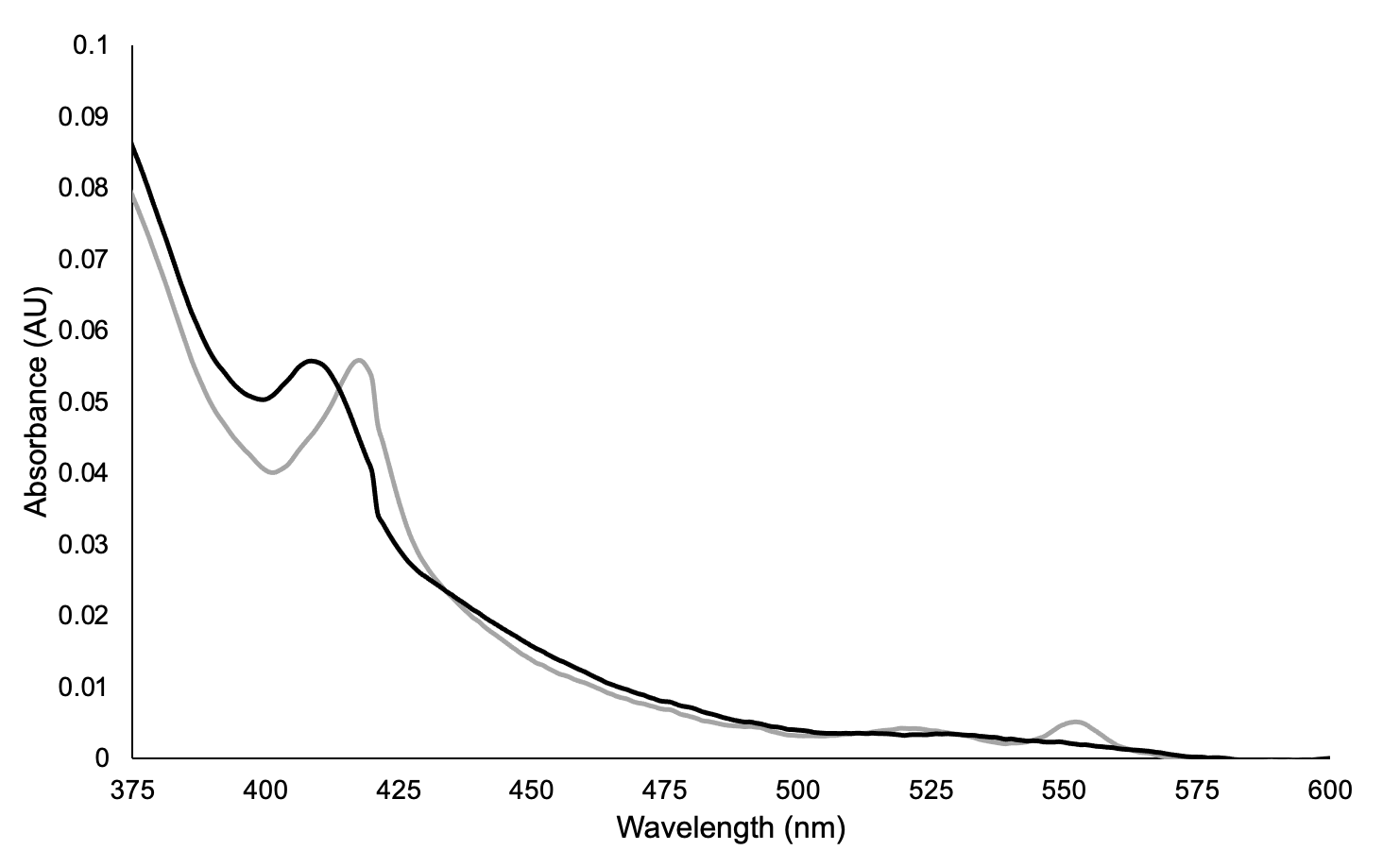
**

**Figure S6: Analytical ultracentrifugation of PgcA monoheme domains.** Sedimentation velocity c(S) analysis of sedimentation velocity data for PgcA monoheme Domain I (**A**), Domain II (**B**), Domain III (**C**) (6 μM) and stoichiometric mixture of all three domains (2 μM each) (**D**), in 20 mM HEPES, 100 mM NaCl pH 7.8 under 129,000 G at 20 °C. Inset top: Raw data sedimentation and fit. Inset middle: 2D difference map. Inset bottom: residual difference plot.

**
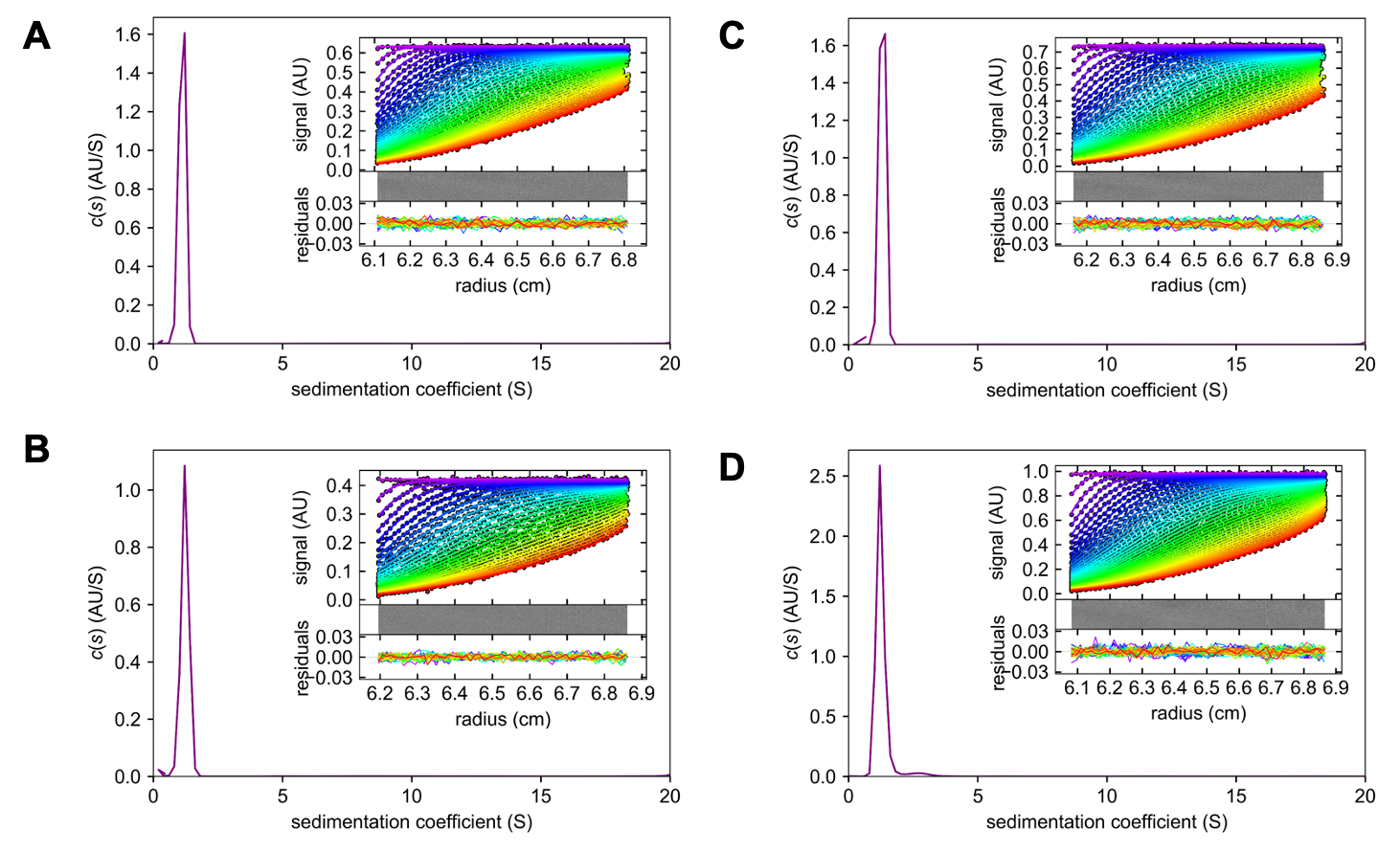
**

**Figure S7: SEC-SAXS signal plots and MultiFoXS model fitting.** (**A**) Triheme PgcA SEC-SAXS traces injected at 10.0 mg mL^-1^. The line shows  the integrated scattering intensity relative to baseline for each scan, open circles are the approximate Guinier R_g_ values calculated for the corresponding scan. (**B**) Log10 intensity plot of subtracted and merged SAXS frames for 10 mg mL^-1^ PgcA. The mean (black) and median (red) buffer subtracted scattering intensity are averaged from selected scans (**C**) Experimental scattering intensity against scattering angle plot (black), MultiFoXS conformation ensemble model-predicted scattering plot (grey) and EOM optimised ensemble model-predicted scattering plot (blue). (**E**) MultiFoXS conformation modelling number fit quality plot.

**
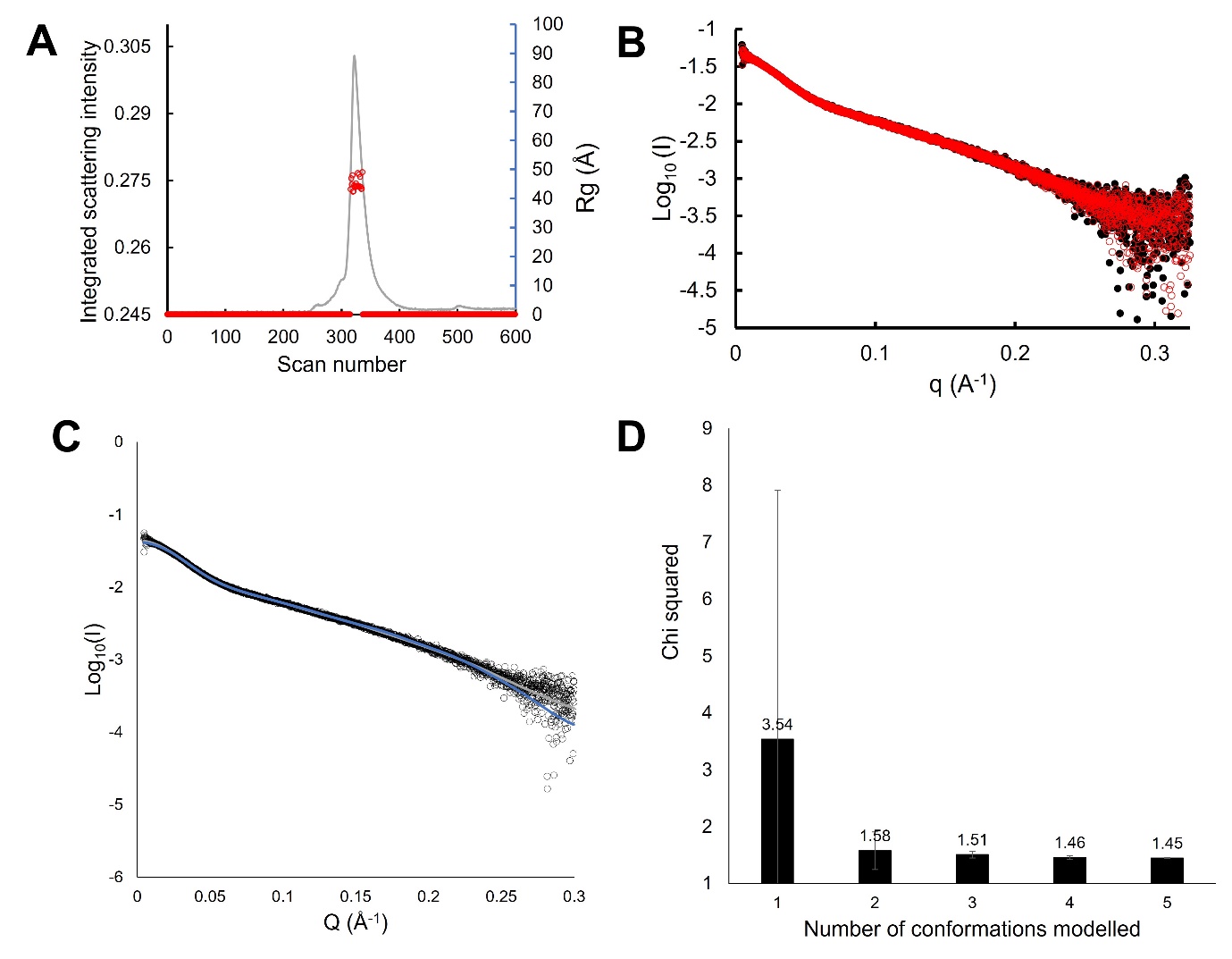
**

**Figure S8: UV-visible spectrum of reduced triheme PgcA and monoheme domains before (colour) and after (black) incubation with iron(III) oxides.**

**
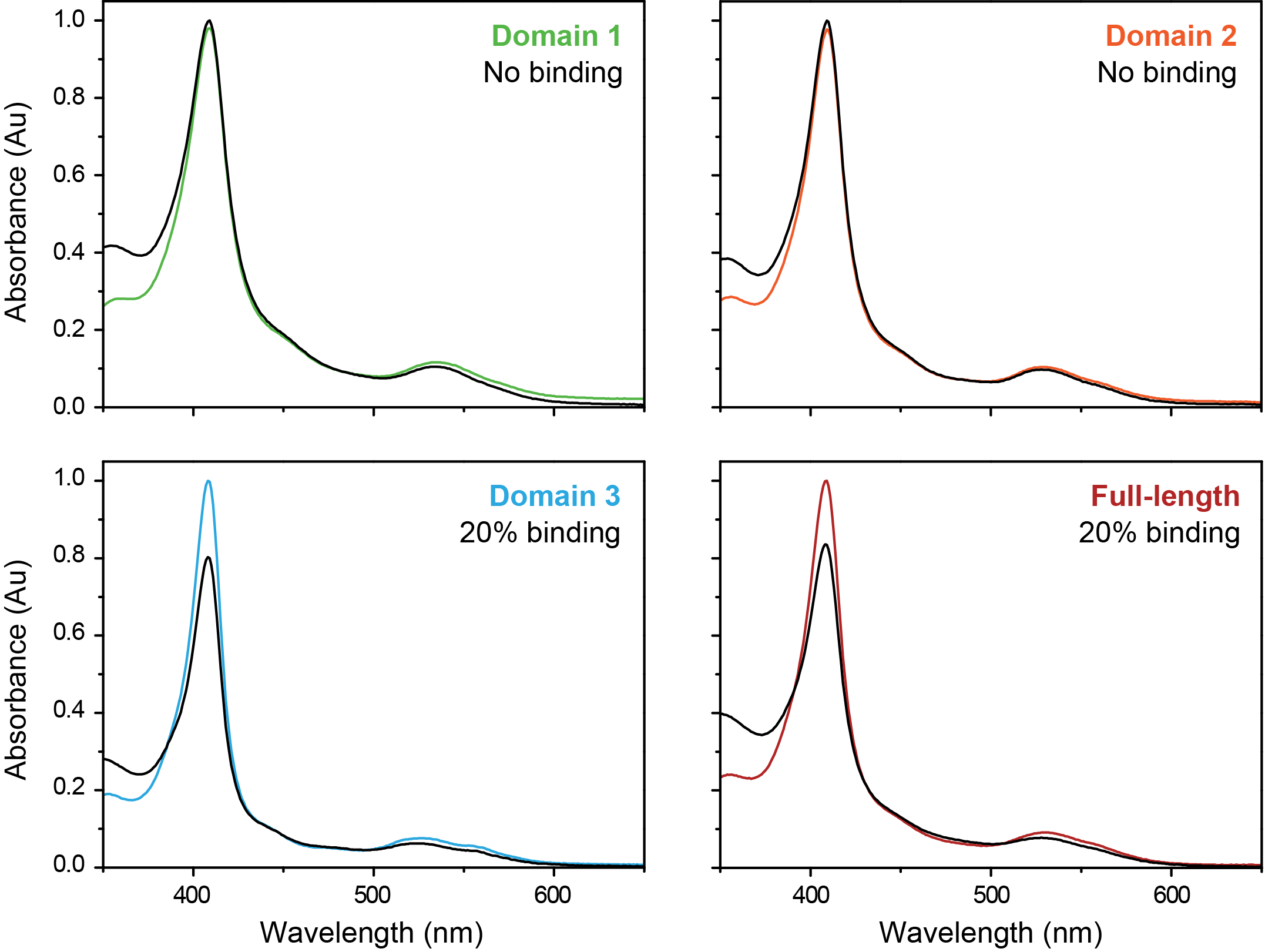
**

**Figure S9: 1D ^1^H-NMR spectra of triheme PgcA.** Spectra were acquired in 32 mM sodium phosphate buffer pH 7.0 at 25 °C.

**
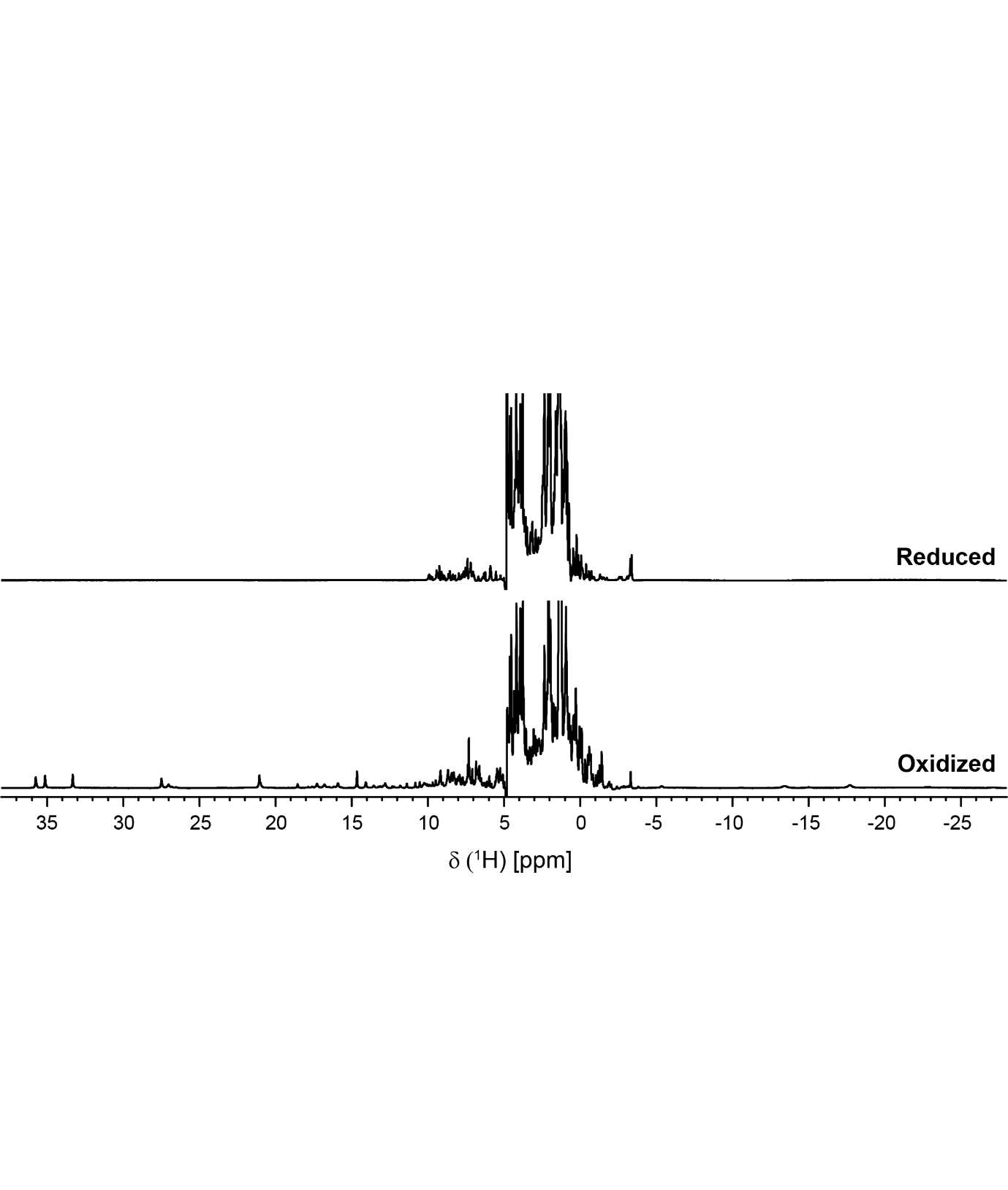
**
